# Does Size Matters? Relationships among Social Dominance and Some Morphometric Traits upon Out-of-Season Reproductive Outcomes in Anestrus Dairy Goats Treated with P4 + eCG

**DOI:** 10.3390/biology9110354

**Published:** 2020-10-24

**Authors:** Santiago Zuñiga-Garcia, César A. Meza-Herrera, Adela Mendoza-Cortina, Carlos Perez-Marin, Noé M. Lopez-Flores, Juan M. Guillén-Muñoz, Gerardo Arellano-Rodriguez, Ulises N. Gutierrez-Guzman, Jorge A. Bustamante-Andrade, Juan R. Luna-Orozco, Francisco G. Véliz-Deras, Nicolás López-Villalobos

**Affiliations:** 1 Universidad Autónoma Agraria Antonio Narro, Unidad Laguna, Periférico Raúl López Sánchez y Carretera a Santa Fe, Torreón, 27054 Coahuila, Mexico; s_zuniga83@hotmail.com (S.Z.-G.); mvz_guillen@hotmail.com (J.M.G.-M.); gveterinarioarellano@gmail.com (G.A.-R.); velizderas@gmail.com (F.G.V.-D.); 2 Facultad de Agricultura y Zootecnia, Universidad Juárez del Estado de Durango, Venecia, 35111 Durango, Mexico; ulisesnoelg@yahoo.com.mx (U.N.G.-G.); abaj_86@hotmail.com (J.A.B.-A.); 3 Universidad Autónoma Chapingo, Unidad Regional Universitaria de Zonas Áridas, Bermejillo, 35230 Durango, Mexico; adela.mendoza@chapingo.uruza.edu.mx (A.M.-C.); noe.lopez.flores@hotmail.com (N.M.L.-F.); 4 Instituto de Estudios de Posgrado, Universidad de Córdoba. Facultad de Veterinaria, 14014 Córdoba, Spain; pv2pemac@uco.es; 5 Centro de Bachillerato Tecnológico Agropecuario No. 1, 27410 Torreón Coahuila, Mexico; jlunaorozco@yahoo.com.mx; 6 Animal and Biomedical Sciences, Institute of Veterinary, Massey University, Palmerston North 442, New Zealand; N.Lopez-Villalobos@massey.ac.nz

**Keywords:** goats, social dominance, anestrous season, estrus induction, morphometric traits, reproductive efficiency

## Abstract

**Simple Summary:**

The possible role of the social rank (R) (i.e., low-LSR, middle-MSR, or high-HSR) in anestrus goats exposed to a P4 + eCG (D) (i.e., 100 or 350 IU) estrus induction protocol (EIP) upon some reproductive response variables was evaluated. Results confirmed that the high social ranked goats amalgamated some fundamental factors to be successful: augmented live weight and corporal measurements, aggressiveness, primacy to food access, and enhanced reproductive outcomes. Such morphometric, behavioral, growth-related, and reproductive advantages shown by the HSR-goats gave evidence to emphasize the need to better comprehend the biological foundation of relevant animal traits, and to be able to define future balanced management and breeding programs. While we still have a fragmentary knowledge regarding the role that social rank, live weight, and morphometric traits play in reproductive success, this study contributes to understanding how social dominance, aligned to morphological and growth related traits, modulates and even determines out-of-season reproductive success.

**Abstract:**

The possible role of the social rank (R) (i.e., low-LSR, middle-MSR, or high-HSR) in anestrus goats exposed to a P4 + eCG (D) (i.e., 100 or 350 IU) estrus induction protocol (EIP) was evaluated. Goats (Alpine-Saanen-Nubian × Criollo; *n* = 70; 25° North) managed under stall-fed conditions were all ultrasound evaluated to confirm anestrous status, while the social rank was determined 30 d prior to the EIP. The response variables included estrus induction (EI, %), duration of estrus (DUR, h), ovulation rate (OR, n), live weight (LW, kg), thoracic perimeter (TP, cm), thoracic diameter (TD, cm), body length (BL, cm), height at withers (HW, cm), beard length (BEA, cm), compactness index (COM, cm), and anamorphosis index (ANA, cm), as affected by R, D, and the R × D interaction were evaluated, while the correlation coefficients among reproductive and morphometric variables were quantified. An R × D interaction (*p* < 0.05) affected the response variables EI, DUR, and OR. While the largest (*p* < 0.05) EI% occurred in the HSR goats, irrespective of eCG (i.e., 100 or 350 IU), both the shortest estrus duration (DUR, h) and the lowest ovulation rate (OR, n) occurred in the LSR + D100 combination, with no differences among HSR and MSR either with D100 or D350. Regarding the LW and morphometric response variables, (i.e., LW, TP, TD, BL, HW, BEA, COM, and ANA) all of them favored either the HSR and MSR groups, with the lowest phenotypic values occurring in the LSR-goats. The EI% was observed to be positively correlated (*p* < 0.05) with DUR (0.71), LW (0.28), TP (0.31), TD (0.34), BL (0.33), HW (0.35), COM (0.23), and ANA (0.23). While DUR was correlated (*p* < 0.05) with TP (0.26) and ANA (0.24), OR demonstrated no-correlation (*p* > 0.05) with any response variable, either reproductive or morphometric. As expected, LW had high correlation coefficients (*p* < 0.01) with TP (0.86), TD (0.88), BL (0.82), HW (0.75), BEA (0.51), COM (0.97), and ANA (0.75). In general, the morphometric variables as a whole demonstrated important correlation coefficients among them (*p* < 0.01), ranging from 0.38 up to 0.84. To estimate the importance of the morphometric differences between social rank upon estrus induction, a principal component (PC) analysis was performed based on the correlation matrix derived from the corporal measurements. The PC1 and PC2 explained 70.3% and 17.6% of the morphometric variation, respectively. The PC1 was a measure of the goat size (i.e., small, medium, large) and its association with estrus occurrence was evaluated using a logistic regression model; the bigger the goat, the increased probability of being in estrus, by up to five times compared to small goats. Our results confirm that the higher social ranked, larger goats amalgamated some fundamental factors to be successful: aggressiveness, primacy to food access, augmented live weight, and corporal size; all of these were able to modulate out-of-season reproductive success in crossbred dairy goats subjected to an estrus induction protocol and managed under stall-fed conditions in Northern Mexico.

## 1. Introduction

Goats are considered a highly sociable species, which is why they form groups both in the wild and in stables [1,2,3]. Goats, when living in society, constantly fight for resources that are scarce, such as food, water, places of rest, and sexual partners [1,4,5]. Therefore, social dominance is established by the agonistic behaviors of goats, such as threats, blows, pushes, pursuits, evasions, and escapes. Such behaviors define whether an individual is dominant or subordinate, depending on the result of the confrontations (win or lose) that they continuously develop [1,6]. The social structure that goats make up is called social hierarchy, where we can observe different social ranks (high, medium, and low), and a dominant animal belongs to the high social rank and a subordinate to the low social rank [4,6,7]. In addition, social rank can influence some sexual parameters of the goat, in effect, goats of the high social rank generally present greater reproductive success than goats of lower social ranks [1,6,7,8].

The Comarca Lagunera (CL), an agro-ecological region located in the arid north of Mexico, has one of the leading concentrations of goats in the Americas, and occupies the first place in goat milk production at national level, generating income under a scheme oriented to the organic production of milk and meat, favoring the producers’ social, economic, and biotic environment [9,10,11]. Such an increase in the regional milk production has been generated because of the massive use of highly specialized genotypes for milk production (i.e., Saanen, Alpine, Anglo-Nubian, and to a lesser extent Toggenburg and Granadina) [12]. Parallel to this significant increase in milk production [9,13], the use of these dairy temperate breeds has also generated a concomitant escalation of the seasonal reproductive activity [14,15]. This discontinuity in the annual reproductive cycle generates, in turn, an evident productive seasonality (i.e., milk and kid-meat) with significant fluctuations between the supply and demand for goat commodities; the latter has generated large economic oscillations, affecting to producers, industrializers, and consumers [16,17]. With the aim of attenuating such discontinuous reproduction–production trends over time, diverse studies have been carried out to modulate reproductive function of females through the use of hormonal treatments, involving the usage of natural progesterone [18], fluorogestone acetate [19], or the medroxyprogesterone acetate [20], routinely complemented with the use of a gonadotropin source, such as human chorionic gonadotropin (hCG) [21] or equine chorionic gonadotropin (eCG) [22]. Currently, short progesterone treatments have been developed, with an interval of 5 to 7 days, reducing the period of exposure of this hormone [22], followed by the use of a gonadotropin to ensure the induction of reproductive activity. Equine chorionic gonadotropin (eCG), applied after the removal of vaginal pessaries, in doses ranging from 250 to 500 IU [19,23,24,25] has given good results. Recently Zúñiga-García et al. (2020) applied a single dose of 25 mg of intramuscular progesterone and 24 h later, administered 100 IU of eCG per goat, generating interesting outcomes regarding the induction of estrous activity, ovulation, and pregnancy [26].

In addition to the mentioned reproductive–productive seasonality observed in various animal species and production systems, the diverse social hierarchies observed among individuals based on the degree of dominance–aggressiveness of some animals, and the level of subordination of others, may also affect reproductive efficiency [27]. Under this scheme, both access to food and reproductive success are not homogeneously exerted by the various group members. Moreover, social dominance, by encouraging better qualitative and quantitative access to food, also generates greater body weights and larger body sizes; such a scenario affords to heavier and bigger animals an enhanced reproductive success [15,28]. This is particularly true under production schemes based on competitive feeding schemes, such as those observed under the zero-grazing or manger feeding systems, regularly used in intensive milk production [29]. Hence, based on previous findings, we hypothesized that an increased social rank status confers a biological advantage, as increased social dominance leads to augmented access to food, improving in turn both live weight and corporal-morphometric values; such a scenario will enhance the out-of-season reproductive success in crossbred anestrous dairy goats subjected to an estrus induction protocol and managed under stall fed conditions. Additionally, we looked to define the principal components which best represent the body size and body condition of goats, and aimed to also identify those principal components that displayed the strongest relationship among estrus induction, social ranking, and body size; this study aims to disentangle such queries.

## 2. Material and Methods 

### 2.1. General

All the experimental procedures, methods, and the managing of the trial experimental units used in this study, were compliant with the guidelines for ethical use, care, and welfare of animals in research at international [30] and national [31] levels, with institutional approval reference number UAAAN-UL-18-3059.

### 2.2. Location, Environmental Conditions, Animals, and Their Management

**Figure 1:**
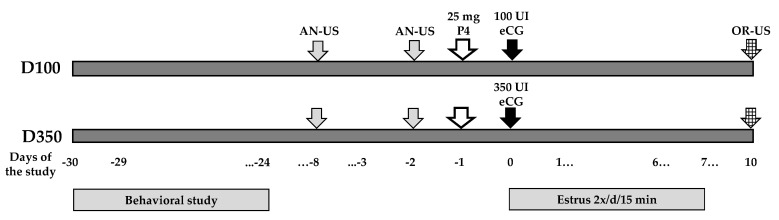
Schematic representation of the experimental protocol. In February, the behavioral study was carried out to define the social ranks; either high (HSR), middle (MSR), or low (LSR) social rank. Then, all goats were exposed to an estrus induction protocol, in order to induce reproductive activity during the natural anestrous season in Northern Mexico (March; 25° North). All goats were primed with progesterone (P4) and received different doses of eCG (100 or 350 IU). Estrus activity was evaluated daily after the application of the eCG doses up to day 7. Transrectal ultrasound (US) scanning were performed on days −8 and −2 to confirm anovulation (AN) as well as on days 10 post-eCG treatment, to assess both ovulatory rate (OR).

This study was conducted in northern Mexico (Comarca Lagunera; 25°51′ N, 103°16′ W, 1190 m), during February and March, the months of the natural anestrous season at this latitude [18,21]. Information regarding the location, environmental conditions, animals, and their management has been previously outlined [7]. Briefly, crossbred-dairy type adult goats (Alpine-Saanen-Nubian x Criollo; *n* = 70, with 2–3 lactations) managed under stall-fed, intensive conditions, were allocated to two uniform groups concerning live weight (LW, 41.85 ± 1.08 kg) and body condition (BCS, 1.87 ± 0.04; scale from 1 to 4) [32]. Experimental groups were lodged in two pens with an area of 80 m^2^ each; fodder was provided three times a day (08:00, 13:00 and 17:00), including alfalfa hay and 200 g per goat/day of commercial concentrate (14% CP). All goats had *ad libitum* access to water and mineral salts. During the pre-trial stage, the anestrous status of goats was established through two trans-rectal ultrasound scans, using a 7.5 MHz human prostate transducer (Aloka 500, MHz linear array; Corometrics Medical Systems, Inc., Wallingford, CT, USA). Prior to the ultrasound scanning, the transducer was lubricated and then inserted into the goat’s rectum to quantify the type of ovarian structures present in both ovaries. Goats with the presence of corpus luteum were discarded from the study. The central activities performed during the experimental period are depicted in Figure 1.

### 2.3. Behavioral Study

One month prior to the treatment group formation (i.e., the application of the eCG), and with the aim of determining the goat’s social rank, a behavioral study was performed, in February as formerly described [1]. The behavioral test was performed at feeding time (08:00, 13:00, and 17:00) during a 60 min period throughout the 7 d pre-trial period. Therefore, the main interactions exerted among breeding female goats were monitored for 180 min d^−1^, for a total of 1260 min (i.e., 21 h) during the whole pre-trial behavioral study. The following behavioral goat-to-goat interactions were documented: bumps, threats, shoves, chases, escapes, and evasions. An interaction was defined when an individual goat displayed dominant behaviors toward a goat that withdrew from the interaction, namely the subordinated goat. The assessed individual behaviors during the feeding time, were considered ad hoc indicators of the aggressive nature of the evaluated animal, while conferring certainty that the high ranked goats had a privileged access to food, as shown at feeding time. Once the agonistic interactions were obtained (i.e., the result of either winning or defeat), an individual success rate (IE) was then calculated: IE = number of individuals able to displace/(number of individuals able to displace + number of individuals displaced). Thereafter, and based on the obtained IE, goats were classified into three social ranks: low (LSR; IE = 0 to 0.33), medium (MSR; IE = 0.34 to 0.66), and high (HSR; IE = 0.67 to 1) [1,6]. Upon confirmation of the anestrus status, and once all goats were social ranked (i.e., LSR, MSR, HSR), they were returned to the pens; fodder was provided three times a day as previously described.

### 2.4. Measurement of Morphometric Variables and Indices according to the Social Rank Status

**Figure 2:**
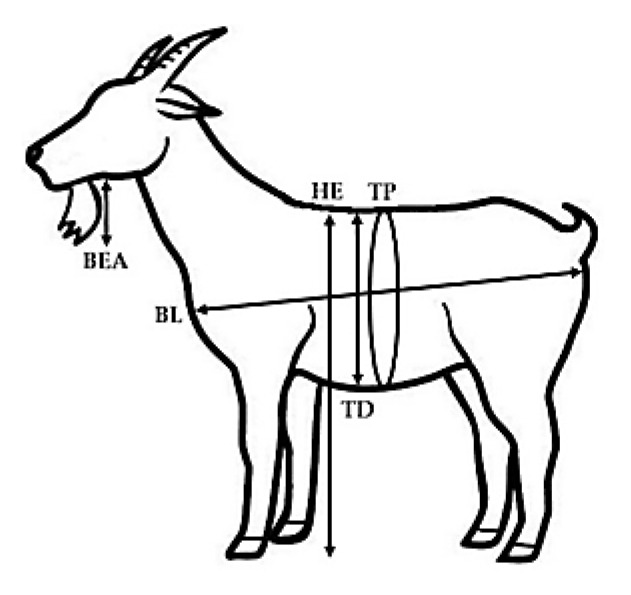
Morphological variables evaluated and their body reference points: TP: thoracic perimeter, cm; TD: thoracic diameter cm; BL: body length, cm; HW: height at withers, cm; BEA: length of beard, cm, in crossbred (Alpine-Saanen-Nubian x Criollo; *n* = 70) dairy goats managed under intensive, stall fed conditions in Northern Mexico (March, 25° North). Data concerning the linear and morphometric measures were collected and elaborated in all goats (n = 70); two morphometric indices were calculated: the compactness index COM = [live weight/height at withers] × 100, and the anamorphosis index ANA = (thoracic perimeter)^2^/height at withers).

Live weight was registered prior to the morning feeding of the behavioral study by using an electronic scale with a capacity of 200 kg and an accuracy of 50 g. Morphological variables were also evaluated at once by a single person. The height at the withers was measured with a zoometric cane, consisting of an adjustable square with a caliper of 1.50 m in height and 1.0 cm measurement accuracy. Body length and thoracic diameter were measured with an adjustable square to the zoometric cane. The thoracic perimeter and the beard length were measured with a calibrated tape measure; live weight and morphometric variables were registered only one time, prior to the behavioral test. The compactness index was calculated considering the formula (live weight/height at withers) × 100. The anamorphosis index was obtained as follows: (thoracic perimeter)^2^/height at withers. The response variables included: estrus induction (EI, %), duration of estrus (DUR, h), ovulation rate (OR, n), live weight (LW, kg), thoracic perimeter (TP, cm), thoracic diameter (TD, cm), body length (BL, cm), height at withers (HW, cm), beard length (BEA, cm), compactness index (COM, cm), and anamorphosis index (ANA, cm). Due to the fact that when defining the goats, social rank status was individually classified, each goat within the eCG dose treatment was defined as the experimental unit. Morphological variables evaluated and their specific body reference points are depicted in Figure 2.

### 2.5. Progesterone + eCG-Based Estrus Induction Protocol Using Two Different eCG Doses (100 and 350 IU)

Thereafter, in the middle of March, all goats received one intramuscular dose of 25 mg of progesterone (Progesvit^®^, Brovel, Mexico). One day later, while the D100 group (*n* = 35) received 100 IU of equine chorionic gonadotropin (eCG) per female (Folligon^®^, Intervet, Mexico), in a simultaneous fashion, the D350 group received 350 IU of eCG per female. Progesterone and eCG were applied intramuscularly; the LSR, MSR, and HSR were randomly located in each eCG dose group. Subsequently, the estrus behavior was tested twice per day (09:00 and 17:00 h) for 15 min, from the day of eCG application, till day 7 of the experimental period (Figure 1). Estrus behavior was defined with the use of 7 sexually active males; in order to prevent sexual intercourse, each buck was aproned. Previously, bucks were subjected to a testosterone hormonal treatment in order to re-activate their sexual behavior and ensure libido [33]. If the female goat remained steady and allowed herself to be mounted by the teaser-buck, estrus commencement was ruled. Afterwards, the male’s apron was detached and the goats from both experimental groups were then exposed to natural mount during the first 12 h after the onset of the estrus. The estrus induction percentage was defined as (number of estrus females/total treated females) × 100. The duration of the estrus was considered the interval between the first and the last mount allowed per goat, additionally, ovulation rate was defined as (total corpus luteum per ovulating goat within group/total goats ovulating within group) × 100.

### 2.6. Statistical Analyses

A first linear model was developed to evaluate the possible relationship of social rank status, and the duration of estrus (DUR, h), live weight (LW, kg), thoracic perimeter (TP, cm), thoracic diameter (TD, cm), body length (BL, cm), height at withers (HW, cm), beard length (BEA, cm), compactness index (COM, cm), and anamorphosis index (ANA, cm), considering the fixed effect of social rank status and the residual error (i.e., Table 1). Regarding percentage and counts variables, since they do not fit normal distribution, estrus induction (EI%), was log^10^ transformed prior to ANOVA to overcome skewness. This second linear model included the fixed effects of social rank status, eCG dose, their interaction, and the residual error (Table 2). Least-squares means and standard errors for each class of social rank status, eCG dose, and the combination of these two factors were computed and used for multiple mean comparisons using Fisher’s least significant difference, as implemented in the LSMEANS option of the PROC GLM of SAS. To quantify the relationship strength between two response variables, while Pearson’s correlation coefficient was used to evaluate quantitative-normally distributed parametric variables, i.e., live weight, Spearman’s correlation coefficient was used to evaluate qualitative-non-normally distributed non-parametric variables, i.e., EI% and OR n [34]. Moreover, in order to compress and reduce the dimensionality to the related nature among the diverse morphometric measurements, a new set of non-correlated variables was generated, although retaining as much as possible the original variation present in the data, and was then analyzed throughout the principal component analysis (PRINCOMP PROC) of SAS. Lastly, a generalized linear mixed model (i.e., logistic regression) was performed to evaluate the association between the animal size classes (i.e., small, medium, big), and the binomial estrus induction variable (i.e., 0, 1) through the PROC GLIMMIX. The sampled animals were split into three groups based on the principal component one (PC1) scores to evaluate the association of this PC1 and estrus induction. The PC1 was a measure of the “size” of the animals, and these groups were defined as small, medium, and big animals. Later on, this association was evaluated using a logistic regression model defined as Logit (Φ*_ij_*) = μ + α*_i_* + e*_ij_*, where Φ*_ij_* is the probability of a doe *j* of size class *i* to be in estrus, μ is general mean, α*_i_* is the effect of size class *i*, and e*_ij_* is the residual error. Odd ratios among size classes were calculated considering small animals as the reference class. Since the female goat social rank status was individually quantified, each goat within the eCG dose was defined as the experimental unit. All the analyses were computed through the procedures of SAS (SAS Inst. Inc. Version 9.4, Cary, NC, USA). The significance level was set at *p* < 0.05.

## 3. Results 

### 3.1. Effect of Social Rank upon the Response Variables

The dependent variables estrus induction (EI, %), duration of estrus (DUR, h), ovulation rate (OR, n), live weight (LW, kg), thoracic perimeter (TP, cm), thoracic diameter (TD, cm), body length (BL, cm), height at withers (HW, cm), beard length (BEA, cm), compactness index (COM, cm), and anamorphosis index (ANA, cm), as affected by the social rank (i.e., HSR, MSR, and LSR), are shown in Table 1. All the response variables, except OR, were affected (*p* < 0.05) by social rank. In general, the LSR-goats showed the lowest values for all the evaluated response variables, either reproductive or morphometric. Furthermore, while the largest EI (*p* < 0.05) occurred in the HSR-goats regarding the MSR and LSR groups (i.e., 96 %, vs. 71 and 56 %), the heavier LW occurred in the HSR and MSR-groups, regarding the LSR goats (i.e., 59.0, 44.0 vs. 31.6 kg, respectively). 

### 3.2. Effect of Social Rank × eCG Dose Interaction upon the Response Variables

A rank x dose interaction (*p* < 0.05) affected the response variables EI, DUR, and OR; the observed values according to HSR, MSR, and LSR as affected by the eCG doses are shown in Table 2. While the largest (*p* < 0.05) EI% occurred in the HSR goats, irrespective of eCG (i.e., 100 or 350 IU), both the shortest estrus duration (DUR, h) and the lowest ovulation rate (OR, units) occurred in the LSR + D100 combination, with no differences among HSR and MSR, either with D100 or D350. Regarding the LW, while the largest values favored (*p* < 0.05) either the HSR and MSR groups, the lowest (*p* < 0.05) phenotypic values occurred in the LSR-goats. 

### 3.3. Correlation Coefficients between the Reproductive and Morphometric Response Variables 

The correlation coefficient values among the morphometric and reproductive response variables (i.e., (EI, %), duration of estrus (DUR, h), ovulation rate (OR, n), live weight (LW, kg), thoracic perimeter (TP, cm), thoracic diameter (TD, cm), body length (BL, cm), height at the withers (HW, cm), beard (BEA, cm), compactness index (COM, units), and anamorphosis index (ANA, units) are shown in Table 3.

The EI% was observed to be positively related (*p* < 0.05) with DUR (0.71), LW (0.28), TP (0.31), TD (0.34), BL (0.33), HW (0.35), COM (0.23), and ANA (0.23). While DUR was correlated (*p* < 0.05) with TP (0.26) and ANA (0.24), OR demonstrated no-correlation (*p* > 0.05) with any of the response variables, either reproductive or morphometric. As expected, LW had high correlation coefficients (*p* < 0.01) with TP (0.86), TD (0.88), BL (0.82), HW (0.75), BEA (0.51), COM (0.97), and ANA (0.75). In general, the morphometric variables as a whole demonstrated important correlation coefficients among them (*p* < 0.01), ranging from 0.38 up to 0.84. With respect to the morphometric indexes COM and ANA, their correlation coefficients with both reproductive and morphologic variables ranged from 0.97 to 0.38; the largest (*p* < 0.01) correlation coefficients occurred between COM and LW (0.97), as well as ANA and TP (0.94).

### 3.4. Principal Component Analyses of the Morphometric Measurements

Principal component analysis (PCA) of morphometric variables, considering the live weight (LW, kg), body condition score (BC, units), height at the withers (HW, cm), body length (BL, cm), thoracic diameter (TD, cm), thoracic perimeter (TP, cm), eigenvalues, total variance (σ, %), and accumulative variance (σ, %) is shown in Table 4. The PCA extracts from the original morphological variation a set of new morphometric variables (i.e., the principal components, PC) that are uncorrelated with one another, and which successively account for maximal amounts of variation among samples. 

**Figure 3:**
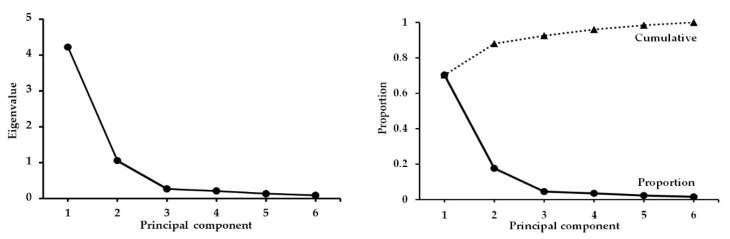
Principal components, the eigenvalues, and the proportional and cumulative variance based on morphometric traits in crossbred dairy goats; plots represent the eigenvalues across the six principal components (**Left panel**), and both proportional and accumulative variance across the six principal components (**Right panel**). Both panels are based on the eigenvectors and eigenvalues generated from correlation matrix among the morphometric values measured from crossbred dairy goats managed under intensive conditions (*n* = 70). Note: more details were previously described in the main body of the text.

Each eigenvector was calculated from an eigenvalue of the correlation matrix of the morphometric values, where the eigenvalues are related to the variance of each PC. That is, the eigenvectors of a correlation matrix determine the direction of the observed variance in a data set; the eigenvector with the highest eigenvalue, that is, the magnitude of the variance, is therefore the PC. The eigenvalues and percentages of the explained variance and cumulative variance for the six principal components based in the morphometric traits are presented in Figure 3. The main outcomes show that the first two PC accounted for 88.01% of the total variance; the remaining four PC explained 11.9% to the total variance.

**Figure 4:**
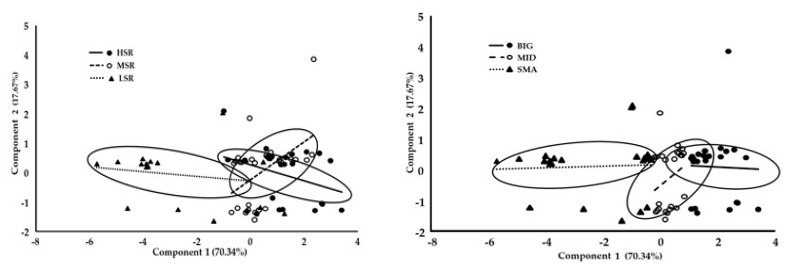
Principal component analysis performed on the morphometric variables in goats; plots represent the first two principle components, considering the scores of the morphological characters representing the morphospace, according to social ranks (i.e., high, HSR; medium, MSR; low, LSR) (**Left panel**) as well as according to body size (i.e., big, BIG; medium, MID; small, SMA) (**Right panel**). Both panels are based on the eigenvectors and eigenvalues generated from the correlation matrix among the morphometric values measured from crossbred dairy goats managed under intensive conditions (*n* = 70). Note: More details were previously described in the main body of the text.

The PCA carried out demonstrated that PC1 and PC2 explained 70.3% and 17.6%, respectively, of the total morphometric variation. The PC analysis revealed that LW, HW, BL, TD, and TP had the highest scores for the first eigenvector, and therefore the PC1 is associated with the size of the animals. With respect to the PC2, this component was more related to the robustness or body condition of the animal, with BCS having the highest PC score (0.96) in the second eigenvector. The six morphometric measures considered in this analysis, revealed that the social ranks (i.e., LSR, MSR, HSR) occupy different morphospaces when examined along the PC1 and PC2 (Figure 4, left panel). In addition, the sampled animals were split into three size groups based on the PC1 scores and these groups were defined as small (SMA), medium (MID), and big (BIG) animals. Therefore, when considering animal size (i.e., SMA, MID, BIG), three identifiable morphospaces among animal size were observed, confirming an important divergence among body sizes (Figure 4, right panel).

Lastly, the association between the animal size classes (i.e., SMA, MID, BIG), and the binomial estrus induction variable (i.e., 0, 1) was evaluated through a logistic regression model. Interestingly, a BIG goat had a five times higher likelihood of exhibiting a positive response to the estrus induction protocol with respect to the smaller goats (*p* < 0.05). Moreover, a significant correlation occurred between social rank status and body size (0.59; *p* < 0.01).

## 4. Discussion

The obtained results support our working hypothesis, in that the largest values of the morphometric response variables, such as thoracic perimeter, thoracic diameter, body length, height at the withers, beard, compactness index, and anamorphosis index were significantly associated to the heavier live weights observed in the HSR-goats, which also had the best estrus induction and estrus duration. Noteworthy is the fact that, regarding the LW and morphometric response variables, all of them favored either the HSR and MSR groups, with the lowest phenotypic values occurring in the LSR-goats. Furthermore, EI% was positively related to LW, while these two were found to be positively correlated with TP, TD, BL, HW, BEA, COM, and ANA. Furthermore, this study defined that while the PC1 was mainly related to body size, the PC2 was primarily associated to the body condition of the goats. In addition, through a logistic regression model it was defined that bigger goats displayed an increased social status, while they were prone to showing an increased estrus activity, up to five times compared with the smaller animals, establishing a strong relationship among corporal size, body condition, social ranking, and reproductive success. This study contributes to the understanding about how social dominance, aligned to morphological and body size related traits, is able to modulate out-of-season reproductive success in goats subjected to an estrus induction protocol.

The optimal reproductive performance obtained by the eCG doses (100 or 350 IU), was due to the double function that this gonadotropin has in its biological activity; it acts mainly as FSH and secondarily as LH. eCG can stimulate the ovary at the follicular level since it can bind to LH receptors on theca cells and FSH receptors on granulosa cells [35]. In addition, the use of eCG after a progestogenic treatment can ensure normal activity and subsequently, a high percentage of pregnancies [19,23,24,25]. The statistical difference in OR observed in our study, could be due to the administered amount of eCG, with the 350 IU dose having a greater stimulation at the ovarian level, promoting an increase in the number and ovulation of preovulatory follicles. This reproductive response is consistent with that of other studies, where similar eCG and hCG doses were used in goats pretreated with intramuscular progesterone [21,26]. On the other hand, a positive effect of the social rank on the reproductive variables was observed, where the HSR goats showed greater reproductive success. While evaluating the possible effect of diverse levels of social dominance in anestrous goats exposed to the cues of the male effect, the largest LH pulsatility and the greatest LH mean concentration occurred in the HSR-goats. Moreover, despite no differences occurring in the number of goats ovulating among social ranks, HSR-goats displayed the greater estrus expression, regarding LSR-goats [8]; the last being similar to our findings. In another study, increases in live weight, average daily gain, follicle number, and diameter also favored the HSR females regarding the LSR-group.

Furthermore, the HSR-females had not only an earlier onset of puberty but also a higher glucose level. Since no differences in LW at puberty occurred between HSR vs. LSR females, such an increased glucose level may explain the amplified productive and reproductive outcomes shown by the HSR-group [36]. Based on these findings [8,36], such an increased glucose level favoring the HSR-females may have been positively related to increases in LW, augmenting in turn both metabolic status and reproductive function in a GnRH-LH dependent fashion, a possible scenario occurring in the HSR-goats of this study. In this respect, although diverse neural and hormonal cues are involved as drivers of the hypothalamic-pituitary-gonadal axis, the common and undisputable initiator involved the activation of GnRH neurons [15]. Whereas the key role of the hypothalamic kisspeptinergic neurons as master regulators not only of GnRH neurons but key elements of the GnRH pulse generator have been proposed. Moreover, the negative feedback of estradiol is exerted by inhibiting the expression of Kiss 1 mRNA at the neurons of the arcuate nucleus, which, in turn, results in gonadotropic inhibition via GnRH; an increase in GnIH activity also appears to play a role in this inhibition [37]. Additionally, the activation of the glutamate receptor is a prerequisite to generate both a pulse and surge pattern of LH release, the glutamatergic transmission being to hypothalamic kisspeptin neurons a critical component [38].

Moreover, a crucial interplay between live weight, metabolic status, and leptin has been proposed; while leptin has been shown to regulate hypothalamic glucose and glutamate transporters [39], several hypothalamic neurons co-express leptin and glutamate receptors [40,41,42], positively influencing synaptic efficacy and ultimately neuronal function. The wide distribution of glutamate receptors in different central nervous system synapses sets this neurotransmitter as a key regulator of a myriad of physiological processes [43,44]. Indeed, while exogenous glutamate administration activated reproductive function in pre-pubertal females, through increases in serum insulin, triiodothyronine [45], and cholesterol [46], in adult anestrus females, glutamate supply also reactivated reproductive function, with concomitant increases in serum insulin and triiodothyronine [47]. Therefore, the possible involvement of glutamatergic and kisspeptinergic neurons as activators of the GnRH neurons in the HSR goats, which also displayed the largest live weights and body measures, could be a conceivably occurring scenario, in the observed out-of-season reproductive outcomes in the HSR-females.

Not only increases in live weight and body size but social dominance have been shown to affect reproductive success, suggesting that females may use social dominance (i.e., aggressiveness) to improve access to food resources [48,49], generating augments in both live weight and body size, while enhancing reproductive outcomes [50]. Since social dominance assures a privileged access to food, while increasing both live weight and energy reserves, it has been proposed that the heavier the mother, the heavier the offspring [51]. Moreover, low weight-undernourished offspring compromise both postnatal growth and puberty onset [52]. Interestingly, under extensive, range based-production systems, such a social dominance, to enhance the access to food resources, could be diminished or even vanish. Certainly, in animal range-based production systems, there might be no selection for individuals striving to achieve higher dominance status, and competition could not be an important strategy to be exerted [29]. Nonetheless, under daily rotational pasture systems, a social hierarchy scheme occurred, with the dominant females spending more time eating grain supplement, whereas subordinate ones spent more time grazing along the paddock [49]. Another key issue when talking about social dominance is aggressiveness, which is an extremely common behavior in all animal species, and whose neurophysiological circuitry and function are quite similar among vertebrates [53,54]. Testosterone (T) receptors are present in diverse hypothalamic neurons; upon aromatization, the newly formed estrogens aligned to a reduction in serotonin at diencephalic level, triggering the aggressive behavior. Aggressiveness will also arise because of the T-increase in the medial amygdala, lateral hypothalamus, and preoptical medial area [53]. Social dominance, testosterone levels, and aggressiveness are tightly aligned. A positive relationship has been reported between HSR ibexes (*Capra Nubiana*) and T-concentrations; the highest T-average concentrations occurred in high ranked males or females. Moreover, the larger the T-value, the greater the increase of the aggression level, and the higher the fertility rate in the HSR-females, with respect to the subordinated ones [54]. On the contrary, a further study proposed that female’s intra-sexual aggression might impact reproductive success in a non-testosterone dependent pathway [55]. Such an interesting strategy may have favored a non-central testosterone role; high T-levels may hinder normal ovulation or engender unfavorable maternal performance.

Regarding the genetic relationships among live weights, body growth, and morphometric measurements, variables such as mature weight (MW), mature weight adjusted for body condition score (AMW), mature height (HT), and body condition (BCS) in ruminants, high estimates of heritability were observed in the first three growth variables (i.e., 0.52, 0.57, 0.71), while BCS was lowly heritable (0.16) [56]. The maternal grandsire line may affect the dominant temperament of the offspring through their mother’s genetic cues, and thereby more aggressive offspring may be further dominant and have, therefore, a higher priority access to food, merging aggressiveness, primacy to food access, augmented corporal growth, and enhanced reproductive outcomes [48]; a such quite interesting scenario may have been occurred in the HSR-goats in our study. Furthermore, noteworthy is the fact that, when evaluating the relationship among dominance, feeding behavior, and methane emissions, the greater the dominance, the longer the feeding time, and the higher the meal frequency. Since the dominant female eats more frequently and for longer, such supremacy at the feeder shown by HSR-females, enhanced not only feed efficiency and corporal growth, but also reduced methane emissions [57]. The last details are of fundamental importance under a scenario marked by global change and climate uncertainty.

From a molecular genetic perspective, growth and (or) morphometric related characters are usually considered to be quantitative traits, controlled by either single genes or several major genes [58]. The copy number variation (CNV) is a normal dissimilarity in the genome sequence of organisms, even larger than the single nucleotide polymorphisms (SNPs) [59]. The CNV mostly enriches the genetic variation of the genome, contributing to augmenting the animal phenotypic diversity [60]. Sequencing analyses of the phosphatidylinositol glycan anchor biosynthesis class Y (*PIGY*) gene have revealed that some regions of this gene overlap with the 28 quantitative trait loci related to sheep economic traits, like muscle density and carcass weight [61]. Some *PIGY*-CNV regions contain large numbers of genes associated with fat metabolism and GTPase activity [62]. Moreover, an important association was detected between CNV of the PIGY gene and diverse growth traits in sheep, such as live weight, chest circumference, and circumference of cannon bone [63]. Additionally, using genome-wide association studies, the *PRDM6* gene affected multiple, apparently non-related inherited traits, emerging as a potentially pleiotropic gene [64]. Thereafter, a 12 bp deletion variant was identified within the *PRDM6* gene in Shaanbei white cashmere yearling goats, associated (*p* = 0.01) with heart girth, cannon circumference, chest depth, chest width, body height, body length, and hip-width; such associations among the deletion variant within the *PRDM6* gene did not occur in older, multiparous animals (i.e., 36 month old) [65]. While these body-growth traits have all been closely related to the economic value in goats, similar morphometric values were also clearly enlarged in the HSR-goats from our study.

**Figure 5:**
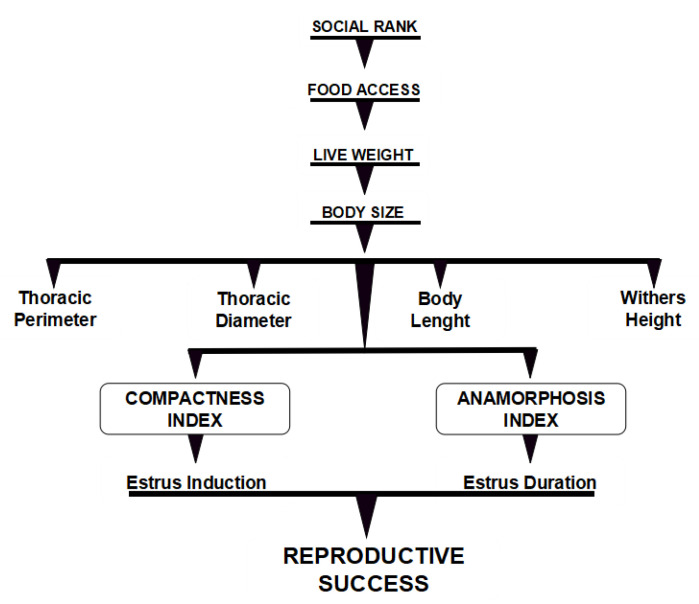
Conceptual diagram modeling the main relationships among social rank, food access, live weight, body size, morphometric values, morphologic indexes, estrus induction, estrus duration, and reproductive success in crossbred (Alpine-Saanen-Nubian × Criollo; *n* = 70) anestrous dairy goats managed under intensive stall fed conditions in Northern Mexico (March, 25° North).

These remarkable research results at molecular-genetic level [59,60,61,62,63,65] make us interconnect such interesting findings, merging them with our research results in order to propose a possible scenario that may help to explain our outcomes. Certainly, the HSR-goats, which also showed the highest live weight (i.e., heavier) and morphometric values (i.e., larger), would have possessed, not only a higher behavioral dominance, but also a genomic advantage (i.e., *PIGY* and *PRDM6* genes expression) with respect to the LSR goats. Undoubtedly, while social dominance ensures access to the best fodder (i.e., quality and quantity), it also warrants not only greater live weights but upholds enhanced reproductive outcomes [7]. Up to this point, the moderate to high heritability of both growth and morphology traits [56], added to the significant correlations among estrus induction, estrus duration, live weight, thoracic perimeter and diameter, and body length observed in our study, suggest a possible interesting scenario. Indeed, according to the latter, such a greater ability to generate heavier and larger values by the HSR-goats can be potentially transmitted to their progeny throughout their genetic makeup across time and space. The latter would assure a privileged hierarchy status, with an increased reproductive performance and growth efficiency, to their next generation youngsters. Moreover, noteworthy is the fact that all of the above can be framed as an enhanced feed efficiency with reduced methane emissions [57], which all together would potentially promote enhanced sustainability, under a cleaner and greener production scheme. Moreover, whereas body size (PC1) explained 70% of the morphometric variation, body condition (PC2) defined 17% of this variation. The number of loci determining size variation is much smaller than those loci governing body shape-condition, either in murine [66] or canine [67] species. Hence, it seems likely that variation in shape or body condition could be a better predictor of genetic differences among social ranks, suggesting that any differentiation in both body size and body shape could indicate differences in the genetic pool among social ranks. Unquestionable, proposing such a possible behavioral, corporal, metabolic, reproductive, genetic, and even eco-friendly scenario in the HSR-goats and their progeny, although enticing, awaits to be scientifically proven. A conceptual diagram of the main findings observed in this study is presented in Figure 5.

## 5. Conclusions

Our results confirm that the high social ranked goats amalgamated some fundamental issues to be successful: augmented live weight and corporal measurements, aggressiveness, primacy to food access, and enhanced reproductive outcomes. Such morphometric, behavioral, size-related, and reproductive advantages shown by the HSR-goats give evidence to emphasize the need to better comprehend the biological foundation of relevant animal traits, in order to define future balanced management, reproductive, and breeding programs. We certainly have a fragmentary knowledge regarding the role that social rank, live weight, and morphometric traits play in reproductive success. Still, the principal component analysis approach allowed a reduction of the number of variables to explain the variance of body conformation (body size and body condition) in crossbred dairy goats. Undoubtedly, the evidence provided by this study, for the first time, contributes to understanding how social dominance, in crossbred dairy goats, aligned to morphological and size related traits (i.e., PC1; size (70.3%) and PC2; body condition (17.6%)), modulate and even determine out-of-season reproductive success (i.e., estrus induction). Therefore, it seems timely while opportune to make reference to an old claim: indisputably, size matters.

## Figures and Tables

**Table 1:** Least-square means ± standard error for estrus induction (EI, %), duration of estrus (DUR, h), ovulation rate (OR, n), live weight (LW, kg), thoracic perimeter (TP, cm), thoracic diameter (TD, cm), body length (BL, cm), height at the withers (HW, cm), beard (BEA, cm), compactness index (COM, units), and anamorphosis index (ANA, units) according to social rank (i.e., LSR, MSR and HSR), and eCG dose (i.e., 100 or 350 IU) in crossbred (Alpine-Saanen-Nubian × Criollo; *n* = 70) dairy goats managed under intensive conditions in Northern Mexico (March, 25° North) ^1,2^.

Variables	Social Rank			
	LSR	MSR	HSR	*p* Value
EI (%)	55 ± 0.09 (10/18) ^b^	71 ± 0.07 (20/28) ^b^	96 ± 0.08 (23/24) ^a^	0.007
DUR (h)	17.3 ± 4.0 ^b^	18.9 ± 3.2 ^b^	29.0 ± 3.5 ^a^	0.004
OR (n)	1.27 ± 0.17 ^a^	1.77 ± 0.13 ^a^	1.58 ± 0.14 ^a^	0.079
LW (kg)	31.6 ± 1.6 ^b^	44.0 ± 1.3 ^a^	49.0 ± 1.4 ^a^	0.001
TP (cm)	83.2 ± 1.5 ^c^	93.5 ± 1.2 ^b^	98.3 ± 1.3 ^a^	0.001
TD (cm)	29.9 ± 0.6 ^b^	34.4 ± 0.5 ^a^	35.5 ± 0.5 ^a^	0.001
BL (cm)	72.7 ± 1.1 ^b^	80.5 ± 0.9 ^a^	81.9 ± 0.9 ^a^	0.001
HW (cm)	64.1 ± 0.9 ^b^	69.8 ± 0.8 ^a^	71.0 ± 0.8 ^a^	0.001
BEA (cm)	3.4 ± 0.9 ^b^	9.4 ± 0.8 ^a^	7.9 ± 0.8 ^a^	0.001
COM (cm)	48.7 ± 2.0 ^b^	63.1 ± 1.6 ^a^	66.1 ± 1.7 ^a^	0.001
ANA (cm)	108.1 ± 3.4 ^c^	125.5 ± 2.7 ^b^	136.6 ± 2.9 ^a^	0.001

^1^ In February, a behavioral study was carried out to define the social ranks; either low (LSR), middle (MSR), or high (HSR) social rank. ^2^ Data concerning the linear and morphometric measures were collected and elaborated in all goats (*n* = 70); two morphometric indices were calculated: the compactness index COM = (live weight/height at withers) × 100, and the anamorphosis index ANA = (thoracic perimeter)^2^/height at withers). ^a,b,c^ Least-square-means without a common superscript within a response variable, are different (*p* < 0.05).

**Table 2:** Least-square means ± standard error for estrus induction (EI, %), duration of estrus (DUR, h), ovulation rate (OR, n), live weight (LW, kg), as affected by the social rank (i.e., LSR, MSR and HSR) x eCG dose (i.e., 100 or 350 IU) interaction in crossbred (Alpine-Saanen-Nubian x Criollo; *n* = 70) dairy goats managed under intensive conditions in Northern Mexico (March, 25° N) ^1^.

Variables	eCG-100				eCG-350	
	LSR	MSR	HSR	LSR	MSR	HSR
EI (%)	38 ± 0.1 (3/8) ^c^	79 ± 0.1 (11/14) ^a,b^	92 ± 0.1 (12/13) ^a,b^	70 ± 0.1 (7/10) ^a,b^	64 ± 0.1 (9/14) ^b,c^	100 ± 0.0 (11/11) ^a^
DUR (h)	7.5 ± 5.9 ^b^	18.9 ± 4.5 ^a^	29.5 ± 4.6 ^a^	25.2 ± 5.3 ^a^	18.9 ± 4.5 ^a^	28.4 ± 5.0 ^a^
OR (n)	0.86 ± 0.3 ^d^	1.2 ± 0.2 ^c,d^	1.2 ± 0.2 ^c,d^	1.6 ± 0.2 ^b,c^	2.3 ± 0.2 ^a^	2.1 ± 0.2 ^a,b^

^1^ In February, a behavioral study was carried out to define the social ranks; either low (LSR), middle (MSR), or high (HSR) social rank. Then, all goats were exposed to an estrus induction protocol, in order to induce reproductive activity during the natural anestrous season in Northern Mexico (March; 25° N). All goats were primed with progesterone (P4) and received different doses of eCG (100 or 350 IU). ^a,b,c,d,^ Least-square-means without a common superscript within a response variable, are different (*p* < 0.05).

**Table 3:** Correlation coefficient matrix between the response variables estrus induction (EI, %), duration of estrus (DUR, h), ovulation rate (OR, n), live weight (LW, kg), thoracic perimeter (TP, cm), thoracic diameter (TD, cm), body length (BL, cm), height at the withers (HW, cm), beard (BEA, cm), compactness index (CON, units), and anamorphosis index (ANA, units) in crossbred (Alpine-Saanen-Nubian x Criollo; *n* = 70) dairy goats managed under intensive conditions in Northern Mexico (March, 25° North) ^1,2^.

Variables	DUR (h)	OR (n)	LW (kg)	TP (cm)	TD (cm)	BL (cm)	HW (cm)	BEA (cm)	CON (cm)	ANA (cm)
EI (%)	**0.717^3^** **0.001**	0.077 0.542	**0.285** **0.016**	**0.311** **0.008**	**0.344** **0.003**	**0.334** **0.004**	**0.359** **0.002**	0.196 0.103	**0.230** **0.054**	**0.238** **0.046**
DUR (h)	1	0.040 0.749	0.234 0.051	**0.260** **0.029**	0.225 0.061	0.179 0.138	0.210 0.080	0.182 0.130	0.212 0.077	**0.244** **0.041**
OR (n)		1	0.174 0.165	0.120 0.341	0.185 0.139	0.127 0.312	0.240 0.053	0.192 0.123	0.133 0.287	0.037 0.767
LW (kg)			1	**0.866** **0.001**	**0.882** **0.001**	**0.828** **0.001**	**0.758** **0.001**	**0.517** **0.001**	**0.970** **0.001**	**0.756** **0.001**
TP (cm)				1	**0.840** **0.001**	**0.748** **0.001**	**0.738** **0.001**	**0.504** **0.001**	**0.812** **0.001**	**0.940** **0.001**
TD (cm)					1	**0.811** **0.001**	**0.768** **0.001**	**0.473** **0.001**	**0.831** **0.001**	**0.715** **0.001**
BL (cm)						1	**0.794** **0.001**	**0.468** **0.001**	**0.755** **0.001**	**0.582** **0.001**
HW (cm)							1	**0.542** **0.001**	**0.584** **0.001**	**0.465** **0.001**
BEA (cm)								1	**0.457** **0.001**	**0.388** **0.001**
CON (cm)									1	**0.773** **0.001**

^1^ In February, a behavioral study was carried out to define the social ranks; either low (LSR), middle (MSR), or high (HSR) social rank. Then, all goats were exposed to an estrus induction protocol, in order to induce reproductive activity during the natural anestrous season in Northern Mexico (March; 25° North). All goats were primed with progesterone (P4) and received different doses of eCG (100 or 350 IU). ^2^ Data concerning the linear and morphometric measures were collected and elaborated in all goats (*n* = 70); two morphometric indices were calculated: the compactness index COM = (live weight/height at withers) × 100, and the anamorphosis index ANA = (thoracic perimeter)^2^/height at withers). ^3^ Bolded and underlined correlation coefficients and probability values, denote statistically significant correlation values (*p* < 0.05).

**Table 4:** Principal component analysis of morphometric variables considering the live weight (LW, kg), body condition score (BC, units), height at the withers (HW, cm), body length (BL, cm), thoracic diameter (TD, cm), thoracic perimeter (TP, cm), eigenvalues, total variance (σ, %), accumulative variance (σ, %) in crossbred (Alpine-Saanen-Nubian x Criollo; *n* = 70) dairy goats managed under intensive conditions in Northern Mexico (March, 25° North).

Principal Component Analysis						
Characters	PC1	PC2	PC3	PC4	PC5	PC6
LW (kg)	0.4595	0.1484	−0.2195	0.1699	0.1087	−0.8233
BC (units)	−0.0267	0.9615	0.2259	0.0687	0.0654	0.1210
HW (cm)	0.4298	−0.1227	0.7675	−0.4495	−0.0406	−0.0850
BL (cm)	0.4441	−0.1524	0.1915	0.6921	0.3657	0.3605
TD (cm)	0.4564	0.0806	−0.2049	0.0947	−0.8240	0.2346
TP (cm)	0.4445	0.0926	−0.4826	−0.5255	0.4115	0.3394
Eigenvalue	4.2205	1.0600	0.2721	0.2121	0.1400	0.0950
Total σ (%)	70.34	17.67	4.54	3.54	2.33	1.58
Accumulative σ (%)	70.3	88.0	92.5	96.0	98.4	100.0
